# ADGRA1 negatively regulates energy expenditure and thermogenesis through both sympathetic nervous system and hypothalamus–pituitary–thyroid axis in male mice

**DOI:** 10.1038/s41419-021-03634-7

**Published:** 2021-04-06

**Authors:** Xiao-Hong Zhang, Ling-Yun Tang, Xi-Yi Wang, Chun-Ling Shen, Wen-Feng Xiong, Yan Shen, Ying-Han Wan, You-Bing Wu, Yi-Cheng Wang, Hong-Xin Zhang, Shun-Yuan Lu, Jian Fei, Zhu-Gang Wang

**Affiliations:** 1 School of Life Sciences and Biotechnology, Shanghai Jiao Tong University, Shanghai, 200240 China; 2 State Key Laboratory of Medical Genomics, Research Center for Experimental Medicine, Rui-Jin Hospital Affiliated to Shanghai Jiao Tong University School of Medicine (SJTUSM), Shanghai, 200025 China; 3 Shanghai Engineering and Technology Research Center for Model Animals, Shanghai Model Organisms Center, Inc., Shanghai, 201318 China; 4 Present Address: Department of Obstetrics and Gynecology, Tang-Du Hospital Affiliated to the Fourth Military Medical University, Xi’an, 710038 China

**Keywords:** Molecular biology, Obesity

## Abstract

Adhesion G protein-coupled receptor A1 (ADGRA1, also known as GPR123) belongs to the G protein-coupled receptors (GPCRs) family and is well conserved in the vertebrate lineage. However, the structure of ADGRA1 is unique and its physiological function remains unknown. Previous studies have shown that *Adgra1* is predominantly expressed in the central nervous system (CNS), indicating its important role in the transduction of neural signals. The aim of this study is to investigate the central function of *Adgra1* in vivo and clarify its physiological significance by establishing an *Adgra1*-deficient mouse (*Adgra1*^*−/−*^) model. The results show that *Adgra1*^*−/−*^ male mice exhibit decreased body weight with normal food intake and locomotion, shrinkage of body mass, increased lipolysis, and hypermetabolic activity. Meanwhile, mutant male mice present elevated core temperature coupled with resistance to hypothermia upon cold stimulus. Further studies show that tyrosine hydroxylase (TH) and β3-adrenergic receptor (β3-AR), indicators of sympathetic nerve excitability, are activated as well as their downstream molecules including uncoupling protein 1 (UCP1), coactivator 1 alpha (PGC1-α) in brown adipose tissue (BAT), and hormone-sensitive lipase (HSL) in white adipose tissue (WAT). In addition, mutant male mice have higher levels of serum T3, T4, accompanied by increased mRNAs of hypothalamus–pituitary–thyroid axis. Finally, *Adgra1*^*−/−*^ male mice present abnormal activation of PI3K/AKT/GSK3β and MEK/ERK pathways in hypothalamus. Overexpression of ADGRA1 in Neuro2A cell line appears to suppress these two signaling pathways. In contrast, *Adgra1*^*−/−*^ female mice show comparable body weight along with normal metabolic process to their sex-matched controls. Collectively, ADGRA1 is a negative regulator of sympathetic nervous system (SNS) and hypothalamus–pituitary–thyroid axis by regulating PI3K/AKT/GSK3β and MEK/ERK pathways in hypothalamus of male mice, suggesting an important role of ADGRA1 in maintaining metabolic homeostasis including energy expenditure and thermogenic balance.

## Introduction

GPCRs compose the largest family of cell-surface mediators of many cellular responses to external stimuli, participating in regulating almost all physiological processes. Dysfunctions of GPCRs are associated with various human diseases, indicating the promising pharmacological targets of GPCRs in treatment and prevention of diseases^1^. GPCRs targeted drugs account for one-third of the drugs in clinical use, but they are only a very small fraction among all the GPCRs^2,3^. Moreover, ~120 members of GPCRs are orphans and their ligands have not been identified, implying the great explored potentiality and utilized value of these GPCRs^4^. Therefore, further comprehensive and intensive study to dissect the physiological function of GPCRs, especially those with unknown function would be beneficial to us for better understanding human diseases and drug development.

ADGRA1 is a member of the adhesion GPCRs which contain long N-termini and multiple domains that are implicated in cell–cell and cell–matrix interactions. Of note, the primary structure of ADGRA1 is peculiar and differs from the other adhesion GPCRs. ADGRA1 is the only member that lacks conserved domain and GPCR autoproteolysis-inducing domain in the extracellular N-terminal region^5,6^. Nevertheless, ADGRA1 exhibits a long C-terminal region containing an end motif ETTV. This motif in tumor endothelial marker 5 (TEM5) protein has been reported to interact with PDZ domain of the human homolog of *Drosophila* discs large tumor suppressor (hDLG) protein during tumor angiogenesis^7^. Therefore, these findings suggest that the interactions between ADGRA1 and other proteins are likely mediated by its conserved C-terminal region and thereby modulating signal transduction.

Previous studies have proven that *Adgra1* was predominantly and widely expressed in the central nervous system (CNS)^5^. Distribution analysis on the functional circuit levels suggests that *Adgra1* may be involved in emotion regulation (amygdala, cortex, and thalamus), learning and memory (hippocampus), and body metabolism (hypothalamus). Moreover, *Adgra1* is well-organized in the layers of brain sections but not in the astrocyte-like scattered patterns^5^, implicating that *Adgra1* is mainly present in neurons rather than astrocytes. Consistently, in the hippocampal neurons, ADGRA1 has been proved to be perfectly co-localized with HOMER1, a marker for the postsynaptic density (PSD)^8^. It is well known that PSD is enriched in scaffolding molecules which anchor neurotransmitter receptors and integrate signals in response to the second messenger cascades activated by the neurotransmitter receptors^9^. Furthermore, most PSD proteins contain PDZ domains, by which they assemble specific proteins into large molecular complexes at defined locations in the cell^10^. Whether ETTV motif in ADGRA1 can interact with PDZ domain of PSD proteins is unknown, but the accumulating evidence makes it reasonable to speculate that ADGRA1 seems to be working in the CNS and controlling the neuronal signal transduction. Importantly, the ADGRA1 is highly conserved in the vertebrate linage, indicating its crucial role in the physiological functions in most vertebrates.

In a word, the *Adgra1* may play an important role in the biological processes and it might be exploited for a therapeutic purpose if its molecular mechanisms were better understood. However, the *Adgra1* is still orphan and the physiological function of *Adgra1* remains unknown. Thus, the aim of this study is to investigate the effect of *Adgra1* on biological process by a knockout mouse model. Our results uncover that *Adgra1*^*−/−*^ male mice exhibit decreased body weight caused by increased energy expenditure, and increased adaptive thermogenesis mediated through SNS and hypothalamus–pituitary–thyroid axis. PI3K/AKT/GSK3β and MEK/ERK pathways are responsible for the abnormal metabolic process. In conclusion, ADGRA1 plays an important role in regulating metabolism homeostasis and negatively regulates energy expenditure and thermogenesis in male mice.

## Results

### Generation of *Adgra1*^*−/−*^ mice

To investigate the physiological function of ADGRA1, we generated an *Adgra1*^*−/−*^ mouse model (Supplementary Fig. 1a–c). The efficient deletion of *Adgra1* was verified by the undetectable expression of both mRNAs and proteins using reverse-transcription PCR, western blotting, and IF analysis (Supplementary Fig. 1d–f).

### *Adgra1* deficiency causes decreased body weight in chow-fed male mice

**Fig. 1:**
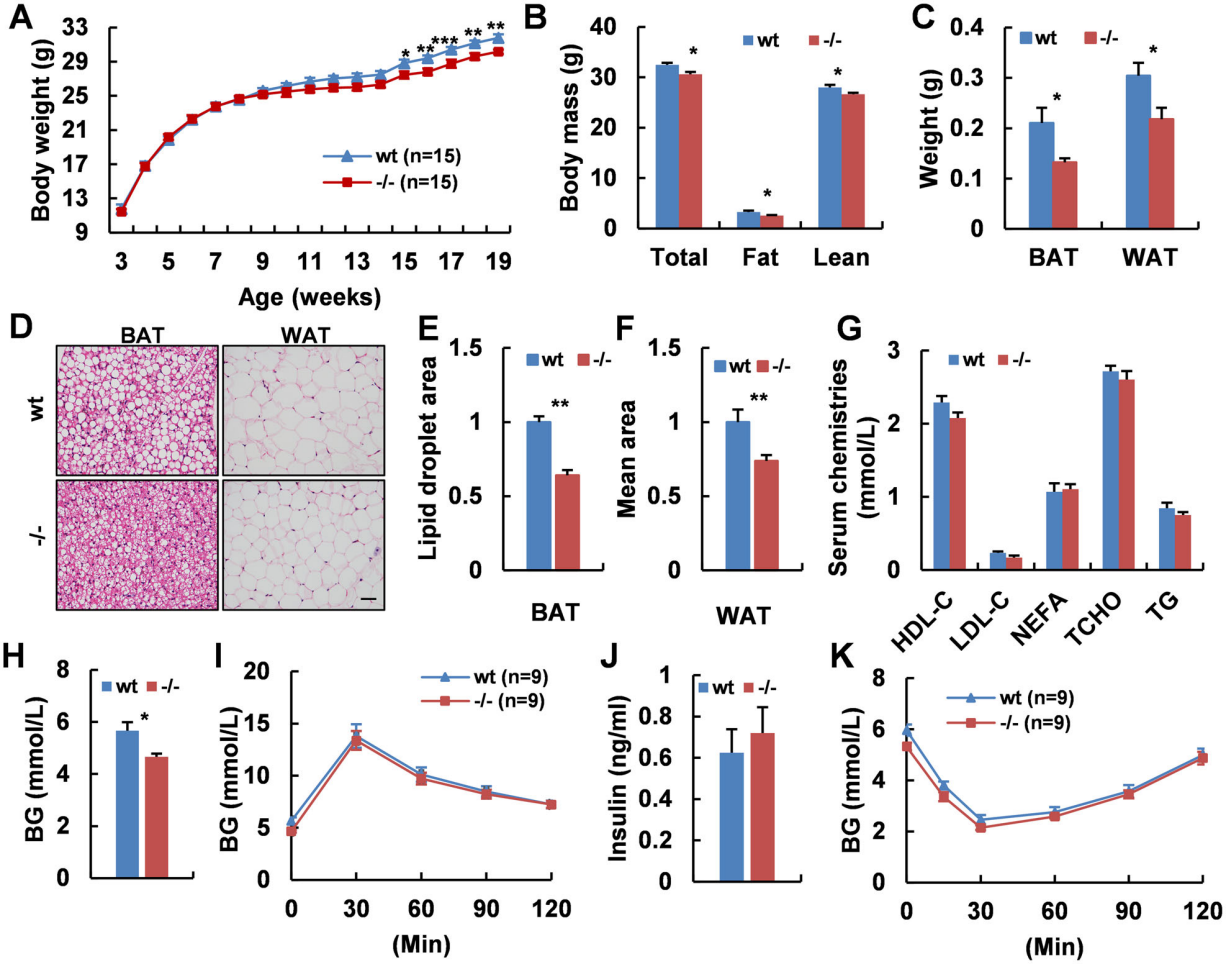
*Adgra1* deficiency causes decreased body weight in chow-fed male mice. **A** Body weight curves of *Adgra1*^*−/−*^ male mice and their littermate controls, *n* = 15/group. **B** Body mass (total, fat, and lean weight) of *Adgra1*^*−/−*^ male and control mice, *n* = 8–9/group. **C** Weights of adipose tissues in *Adgra1*^*−/−*^ male and control mice, *n* = 5/group. **D** Representative images of HE stained BAT, and WAT from *Adgra1*^*−/−*^ male and control mice. Scale bar, 50 μm, *n* = 5/group. **E** Quantify of the lipid droplet-positive area in the BAT, *n* = 5/group. **F** Quantify of the mean area of the adipocytes in the WAT, *n* = 5/group. **G** Serum chemistries in *Adgra1*^*−/−*^ male and wt mice in fed state, *n* = 9/group. **H** Basal blood glucose levels after fasted overnight of *Adgra1*^*−/−*^ male and control mice, *n* = 9/group. **I** GTT in *Adgra1*^*−/−*^ male and wt mice, *n* = 9/group. **J** Serum insulin levels of *Adgra1*^*−/−*^ male and control mice, *n* = 9/group. **K** ITT in *Adgra1*^*−/−*^ male and wt mice, *n* = 9/group. BG blood glucose. *p < 0.05, **p < 0.01, ***p < 0.001 vs. controls. Data represent mean ± S.E.M.

Mice were fed standard chow and their body weight was monitored once a week. Surprisingly, *Adgra1*^*−/−*^ male mice exhibited significantly less weight gain than their littermate controls from 15 weeks on (Fig. 1A). Consistently, *Adgra1*^*−/−*^ male mice displayed a significant reduction in fat and lean mass, decreased weights of adipose tissues, and decreased lipid accumulation in adipocytes (Fig. 1B–F). Though the serum chemistry analysis showed no significant differences between the two genotypes, the lipoprotein cholesterol and triglycerides (TG) in male mutant mice were slightly less than that in controls (Fig. 1G). Moreover, the basal glucose level was lower in *Adgra1*^*−/−*^ male mice, but the glucose tolerance was intact (Fig. 1H, I). Meanwhile, both serum insulin levels and insulin tolerance test were normal (Fig. 1J, K). Here, female mice were also monitored, but they exhibited comparable body weight and similar body composition between two genotypes (Supplementary Fig. 2a, b). Consistently, serum chemistry examination, histology analysis of adipose tissues, and glucose homeostasis test all showed no obvious changes (Supplementary Fig. 2c–f). Taken together, the evidence implicated that *Adgra1* deficiency caused abnormal metabolism in male but not in female mice.

### *Adgra1* deficiency has no obvious effect on the histology of major organs and serum chemistry in mutant male mice

We examined the gross anatomy of organs including brain, heart, liver, spleen, lung, and kidney. Obviously, no apparent abnormalities in shapes and weights of main organs were found between *Adgra1*^*−/−*^ male mice and their littermate controls (Supplementary Figs. 3a–c and 4a–c). Meanwhile, histological analyses by HE staining revealed no structure alterations in the brain (Supplementary Fig. 3d). In addition, no significant differences were observed in the serum parameters for live function, cardiovascular function, and kidney function by the serum biochemical analysis (Supplementary Fig. 4d–f).

### *Adgra1* is specifically expressed in the CNS

The tissue expression profile revealed that *Adgra1* was exclusively and dominantly detected in brains both in mouse and human (Supplementary Fig. 5a, b). We further investigated the exact distribution in CNS and found that *Adgra1* was highly expressed in the cortex, hypothalamus, hippocampus, cerebellum and spinal cord. An intriguing finding was that expression of *Adgra1* in hypothalamus was markedly higher than that in the other brain regions, raising the rationality that *Adgra1* plays an important role in regulating body metabolism. Furthermore, we detected *Adgra1* mRNA levels in the key metabolic tissues including pituitary, thyroid, and adipose tissues, and found that the expression of *Adgra1* was rarely detected in these metabolic tissues other than hypothalamus (Supplementary Fig. 5a). Thus, it is reasonable to speculate that any involvement of *Adgra1* in body metabolism is possibly mediated through hypothalamus. In addition, ADGRA1 was identified to co-localize with NEUN-positive neuron cells rather than GFAP-positive astrocytes by IF on sections of brain regions (Supplementary Fig. 5c). The localization of ADGRA1 was on the membrane by visualizing EGFP signals from ADGRA1-EGFP fusion protein and EGFP protein only (Supplementary Fig. 5d). IF staining showed the comparable CNS cells between *Adgra1*^*−/−*^ male mice and their littermate controls (Supplementary Figs. 6–9, a–d).

### *Adgra1*^*−/−*^ male mice present hypermetabolic activity

**Fig. 2:**
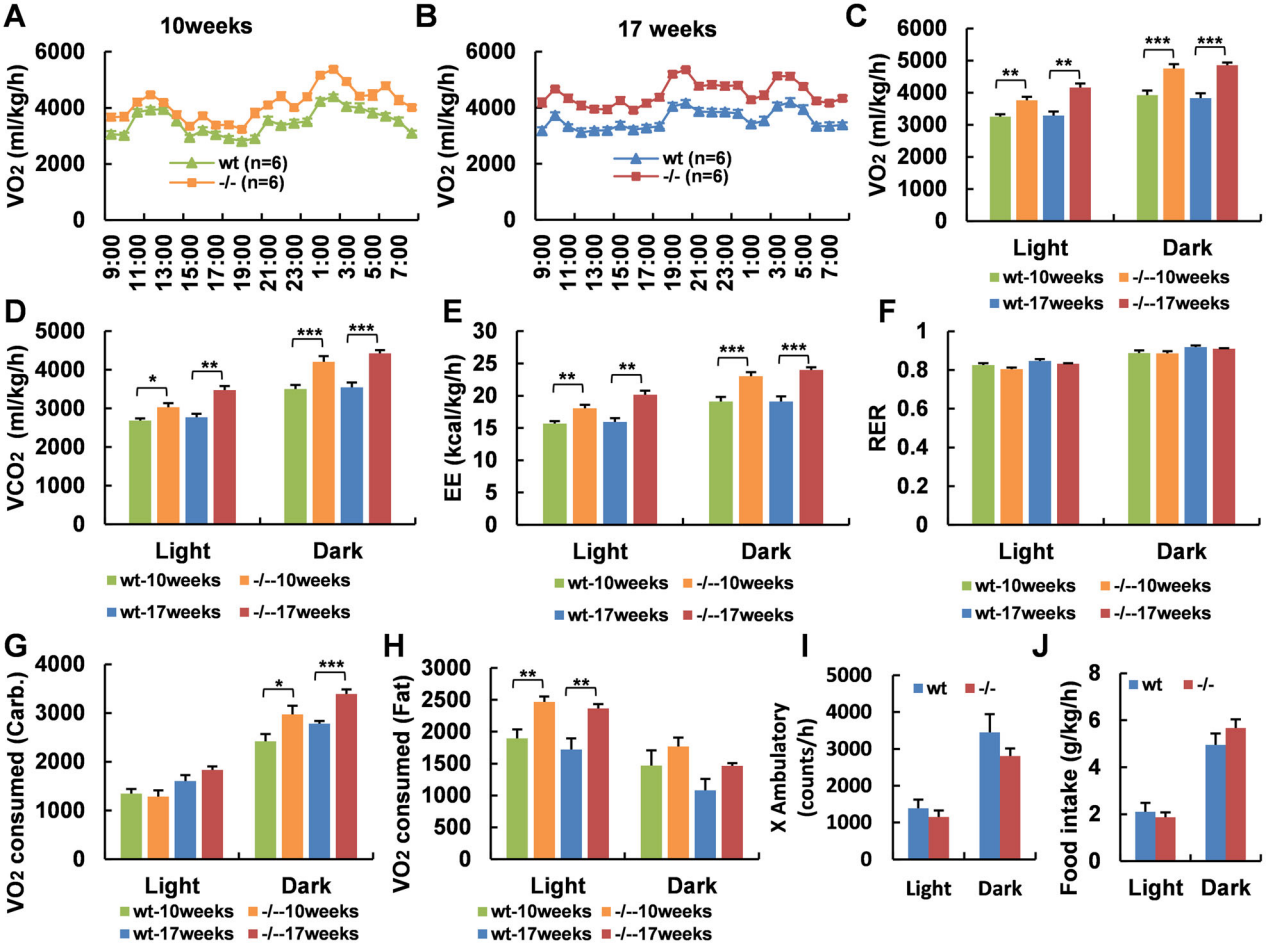
*Adgra1* deficiency induces increased energy expenditure in male mice. **A**–**F** At the age of 10 weeks and 17 weeks, the metabolic cage studies assess O_2_ consumption in curve diagram (**A** for 10 weeks, **B** for 17 weeks) and histogram (**C**), CO_2_ production (**D**), energy expenditure (**E**), and RER (**F**) during light and dark of *Adgra1*^*−/−*^ male and control mice, *n* = 6/group. EE, energy expenditure. **G**, **H** O_2_ consumption for carbonhydrate (**G**) and fat (**H**) during light and dark, *n* = 6/group. **I** Locomotor activities in both genotypes at the age of 17 weeks, *n* = 6/group. **J** Food intake in both genotypes at the age of 17 weeks, *n* = 6/group. *p < 0.05, **p < 0.01, ***p < 0.001 vs. controls. Data represent mean ± S.E.M.

**Fig. 3:**
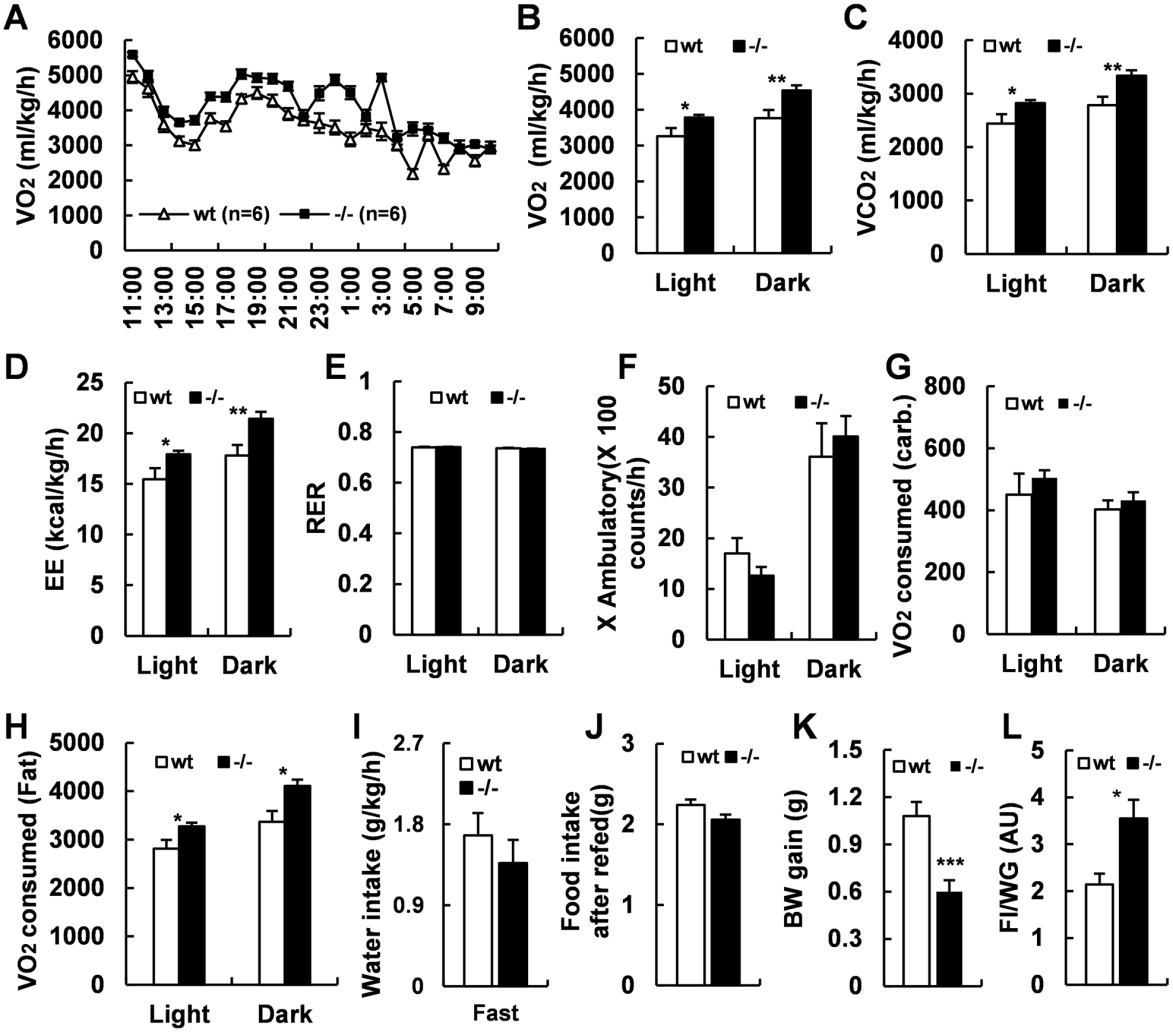
Basal metabolic rate is increased in fasted *Adgra1*^*−/−*^ male mice. **A**–**F** At the age of 17 weeks, the metabolic cage studies assess O_2_ consumption in curve diagram (**A**) and histogram (**B**), CO_2_ production (**C**), energy expenditure (**D**), RER (**E**), and locomotor activities (**F**) during light and dark of fasted *Adgra1*^*−/−*^ male and control mice, *n* = 6/group. EE energy expenditure. **G**, **H** O_2_ consumption for carbonhydrate (**G**) and fat (**H**) during light and dark, *n* = 6/group. **I** Water intake in fasted states, *n* = 6/group. **J** Food intake after refed for 24 h, *n* = 15/group. **K** Body weight gain after refed for 24 h, *n* = 15/group. **L** Ratio of food intake to body weight gain in fasted-refed male mice, *n* = 15/group. *p < 0.05, **p < 0.01 vs. controls. Data represent mean ± S.E.M.

To further understand how *Adgra1* deficiency affected the less body weight gain, we performed metabolic cage studies in both genotypes at the age of 10 weeks when their body weights were comparable and at the age of 17 weeks when *Adgra1*^*−/−*^ male mice were significantly thinner than their littermate controls. The results showed that oxygen consumption, carbon dioxide production, and energy expenditure in *Adgra1*^*−/−*^ male mice were significantly elevated at the age of 10 weeks, excluding the secondary effect on the metabolic phenotypes of *Adgra1*^*−/−*^ male mice. Of note, the alterations of metabolic parameters reached higher levels at the age of 17 weeks, pointing to a progressive activation of energy expenditure in the loss of ADGRA1, and making it clear that the decreased body weight gain in *Adgra1*^*−/−*^ male mice was a result of gradual accumulation of hypermetabolic activity (Fig. 2A–E). Though RER was equivalent in *Adgra1*^*−/−*^ male mice compared to their littermate controls (Fig. 2F), we deeply explored the exact destination of oxygen consumption based on RER and VO_2_ according to the literature^11^. Obviously, *Adgra1*^*−/−*^ male mice preferred to consuming O_2_ for fat oxidation in light and for carbohydrate oxidation in dark at both ages (Fig. 2G, H), which could be explained by feeding habits of mice that they usually take food at night. Ambulating locomotor activities and food intake were unchanged at the age of 17 weeks (Fig. 2I, J) as well as at the 10 weeks (data not shown). Next, we removed the food and measured the basal metabolic rate. Consistently, *Adgra1*^*−/−*^ male mice exhibited persistently hypermetabolic activity in fasted status (Fig. 3A–F). According to RER and VO_2_, oxygen consumption for fat was enhanced in both light and dark, whereas oxygen consumption for carbohydrate was similar all along (Fig. 3G, H), confirming that *Adgra1*^*−/−*^ male mice consumed more oxygen for carbohydrate oxidation when they took in food and consumed more oxygen for fat oxidation when they did not need food or the food was taken away. Consequently, these combined results revealed that the energy expenditure is consistently higher in *Adgra1*^*−/−*^ male mice regardless of the feeding behaviors. But whether appetite control is involved in this process was unclear. Thus, we returned the food back to the fasted mice and monitored the refeeding behaviors. As shown, food intake was similar after fasted (Fig. 3J), suggesting the unaffected appetite in *Adgra1*^*−/−*^ male mice. However, the less weight gain and the higher ratio of food intake to body weight gain proved that the ingested fuels in *Adgra1*^*−/−*^ male mice were used for expenditure rather than storage (Fig. 3K, L). But in female mice, metabolic cage studies showed the energy metabolism was unaffected (Supplementary Fig. 10a–i). Taken together, these data demonstrate that decreased body weight is associated with the increased basal metabolic rate and the phenotype only occurred in adulthood of male but not female mice.

### Adaptive thermogenesis is increased in *Adgra1*^*−/−*^ male mice

**Fig. 4:**
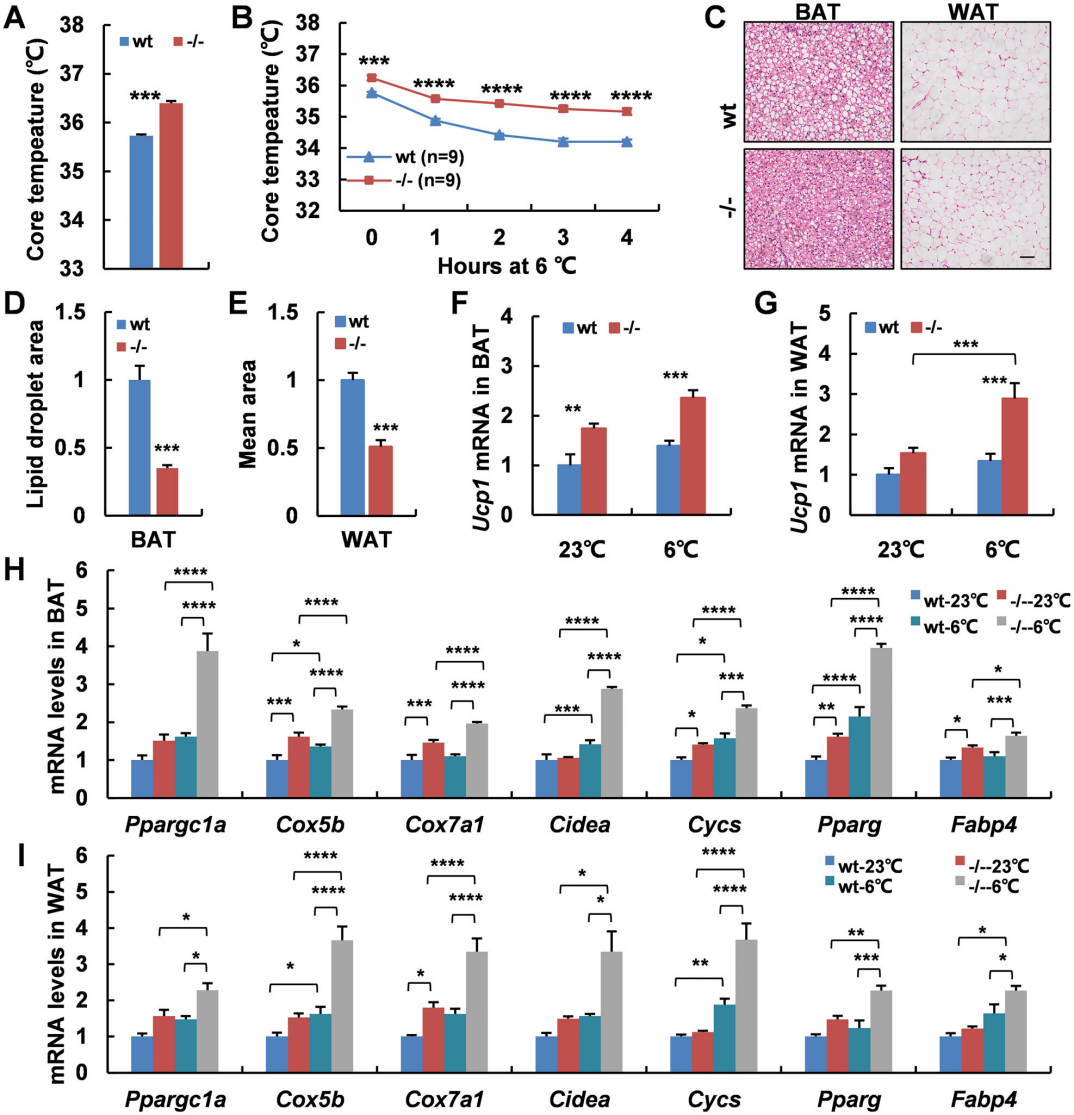
Adaptive thermogenesis is increased in *Adgra1*^*−/−*^ male mice. **A** Rectal temperature of *Adgra1*^*−/−*^ male and control mice, *n* = 9/group. **B** Rectal temperature of *Adgra1*^*−/−*^ male and control mice when they are exposed to cold (6 °C) for 4 h, *n* = 9/group. **C** Representative images of HE stained BAT, and WAT from *Adgra1*^*−/−*^ male and control mice in cold environment. Scale bar, 50 μm, *n* = 5/group. **D** Quantify of the lipid droplet-positive area in the BAT, *n* = 5/group. **E** Quantify of the mean area of the adipocytes in the WAT, *n* = 5/group. **F** *Ucp1* mRNA levels of BAT in room temperature and cold environment, *n* = 5/group. **G** *Ucp1* mRNA levels of WAT in room temperature and cold environment, *n* = 5/group. **H** mRNA levels of thermogenic genes of BAT in room temperature and cold environment, *n* = 5/group. **I** mRNA levels of thermogenic genes of WAT in room temperature and cold environment, *n* = 5/group. *p < 0.05, **p < 0.01, ***p < 0.001, ****p < 0.0001 vs. controls. Data represent mean ± S.E.M.

To further understand the underlying mechanisms for the increased energy expenditure, we assessed the core body temperature of mice at room temperature and observed the mean rectal temperature in *Adgra1*^*−/−*^ male mice was higher than that of their littermate controls (Fig. 4A). When acutely exposed to 6 °C for 4 h, *Adgra1*^*−/−*^ male mice still presented higher rectal temperature, meaning cold-induced adaptive thermogenesis was increased (Fig. 4B). Meanwhile, cold stimulation reduced the lipid droplets and the difference was significant (Fig. 4C–E). Consistently, Ucp1, a thermogenic marker in adipocytes, was activated at room temperature and cold strengthened its expression in *Adgra1*^*−/−*^ male mice (Fig. 4F, G). Similar alterations were also observed in other thermogenic genes in both BAT and WAT (Fig. 4H, I). Collectively, ADGRA1 deficiency increases the adaptive thermogenesis in male mice.

### SNS is activated in *Adgra1*^*−/−*^ male mice

**Fig. 5:**
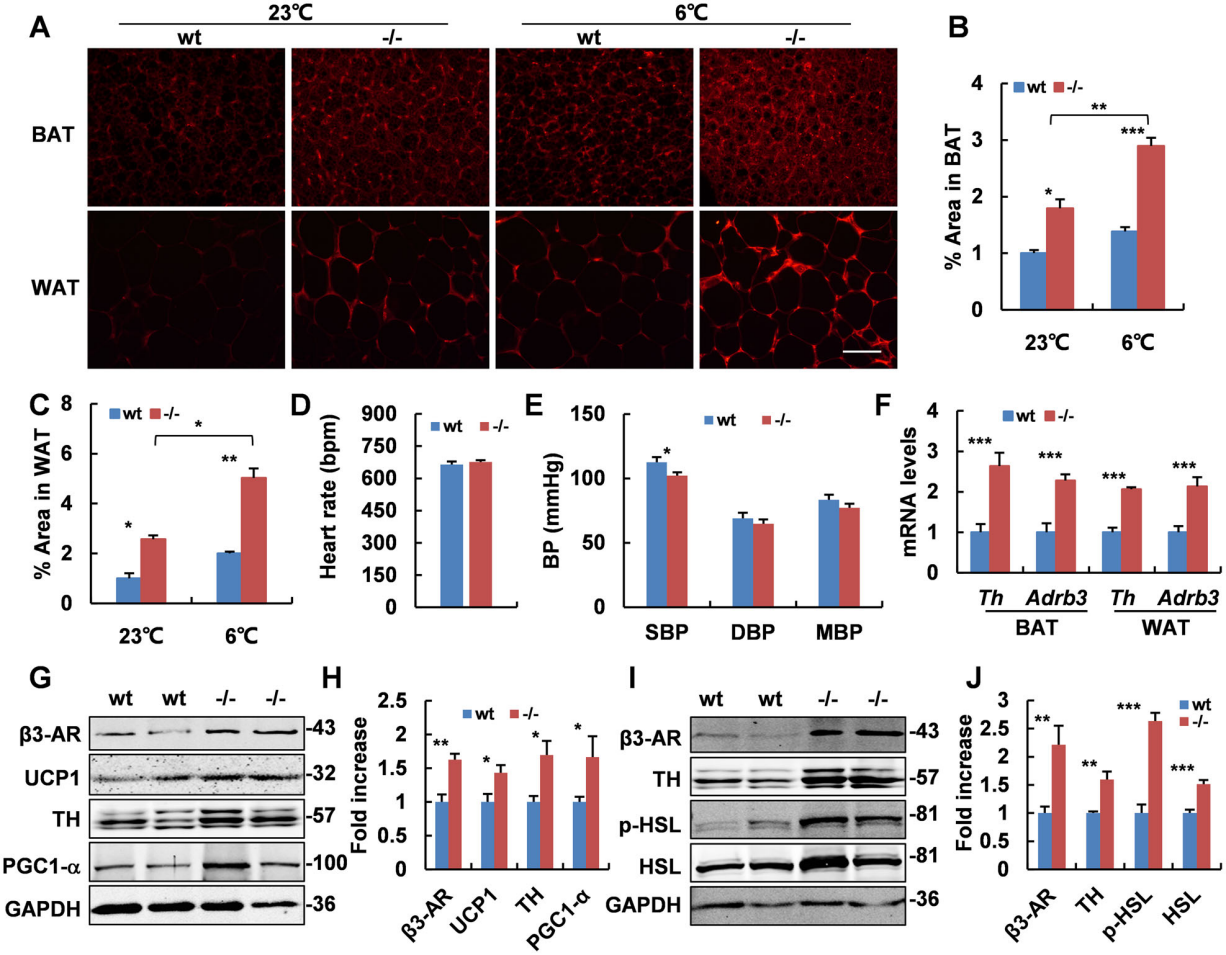
SNS is activated in *Adgra1*^*−/−*^ male mice. **A** Representative images of IF of TH in BAT and WAT in room temperature and cold environment. Scale bar, 50 μm. **B** Quantitative analysis of positive areas of TH in BAT, *n* = 5/group. **C** Quantitative analysis of positive areas of TH in WAT, *n* = 5/group. **D** Heart rate in male mice of both genotypes, *n* = 9/group. **E** Blood pressure in male mice of both genotypes, *n* = 9/group. SBP systolic blood pressure, DBP diastolic blood pressure, MBP mean blood pressure. **F** mRNA levels of *Th* and *Adrb3* in BAT and WAT assessed by real-time PCR, *n* = 5/group. **G** Protein levels in BAT of *Adgra1*^*−/−*^ male and control mice analyzed by western blot. GAPDH is used as a loading control. **H** Quantitative analysis of relative intensities of proteins. *n* = 5/group. **I** Protein levels in WAT of *Adgra1*^*−/−*^ male and control mice analyzed by western blot. GAPDH is used as a loading control. **J** Quantitative analysis of relative intensities of proteins, *n* = 5/group. Western blot analysis using anti-TH antibodies detects a band at ~57 kDa, at a position where the TH is expected to migrate, with two nonspecific bands running above and below. *p < 0.05, **p < 0.01, ***p < 0.001 vs. controls. Data represent mean ± S.E.M.

BAT thermogenesis is well considered as a response to the stimulation of SNS. Thus, we assessed the activity of SNS through analyzing the levels of TH. As expected, TH was much more abundant in adipocytes of *Adgra1*^*−/−*^ male mice both at room temperature and cold enhanced its activation by IF (Fig. 5A–C). To investigate how TH regulated the SNS activity, we tested the related genes involved in this process. As shown, TH and β3-AR were both increased in mRNAs and proteins, as a result, the downstream cascades in thermogenic process were activated (Fig. 5F–J). Therefore, these combined results demonstrate that abolition of *Adgra1* increases sympathetic outflow into adipocytes, enhancing adaptive thermogenesis in male mice. Given that cardiac SNS activity was also regulated by hypothalamus via sympathetic premotor neurons, we detected the heart rate (Fig. 5D) and blood pressure (Fig. 5E) of adult male mice, but no differences were observed. The unaffected cardiac SNS activity may be a consequence of compensation effects in vivo. In contrast, *Adgra1*^*−/−*^ female mice exhibited comparable core temperature at both room and cold conditions (Supplementary Fig. 11a, b), and no significant difference of the SNS activity was observed (Supplementary Fig. 11c–g).

### *Adgra1*^*−/−*^ male mice exhibit mild central hyperthyroidism

**Fig. 6:**
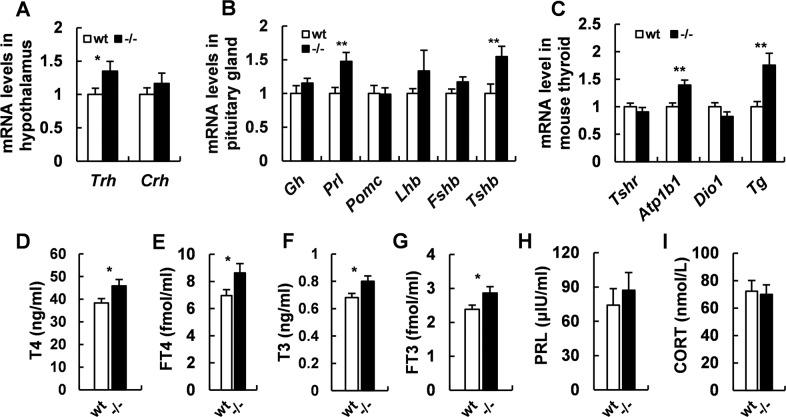
*Adgra1*^*−/−*^ male mice exhibit mild hyperthyroidism. **A** mRNA levels of *Crh* and *Trh* in hypothalamus of male mice assessed by real-time PCR, *n* = 5/group. **B** mRNA levels of trophic hormones in pituitary gland of male mice assessed by real-time PCR, *n* = 5/group. **C** mRNA levels of thyroid hormones-related genes in thyroid of male mice assessed by real-time PCR, *n* = 5/group. **D**–**I** Serum hormones levels of *Adgra1*^*−/−*^ male and control mice. **D** T4 levels, *n* = 14/group. **E** Free T4 levels, *n* = 7–9/group. **F** T3 levels, *n* = 12/group. **G** Free T3 levels, *n* = 7–9/group. **H** PRL levels, *n* = 4/group. **I** Corticosterone levels, *n* = 13–14/group. *p < 0.05, **p < 0.01 vs. controls. Data represent mean ± S.E.M.

hypothalamus–pituitary–thyroid axis participates in the body growth and development by maintaining body energy homeostasis^12,13^. Therefore, we found that mRNAs of thyrotropin-releasing hormone (*Trh*) in hypothalamus and thyroid-stimulating hormone-β subunit (*Tshb*) in pituitary (Fig. 6A, B) were elevated in *Adgra1*^*−/−*^ male mice. Meanwhile, thyroglobulin (*Tg*) and Na^+^/K^+^-ATPase (*Atp1b1*), involving in the synthesis and transportation of THs, were markedly upregulated in thyroid (Fig. 6C). Moreover, serum THs, including T4 and T3, were also increased in both total and free forms (Fig. 6D–G). Serum PRL levels were unchanged though the mRNA levels were increased (Fig. 6B, H). The similar mRNA levels of *Crh* and comparable serum levels of corticosterone implied the normal function of hypothalamus–pituitary–adrenal gland axis in male knockout mice (Fig. 6A, I). All above, *Adgra1*^*−/−*^ male mice exhibit mild central hyperthyroidism.

### Activated PI3K/AKT/GSK3β and MEK/ERK pathways in the hypothalamus of *Adgra1*^*−/−*^ male mice

**Fig. 7:**
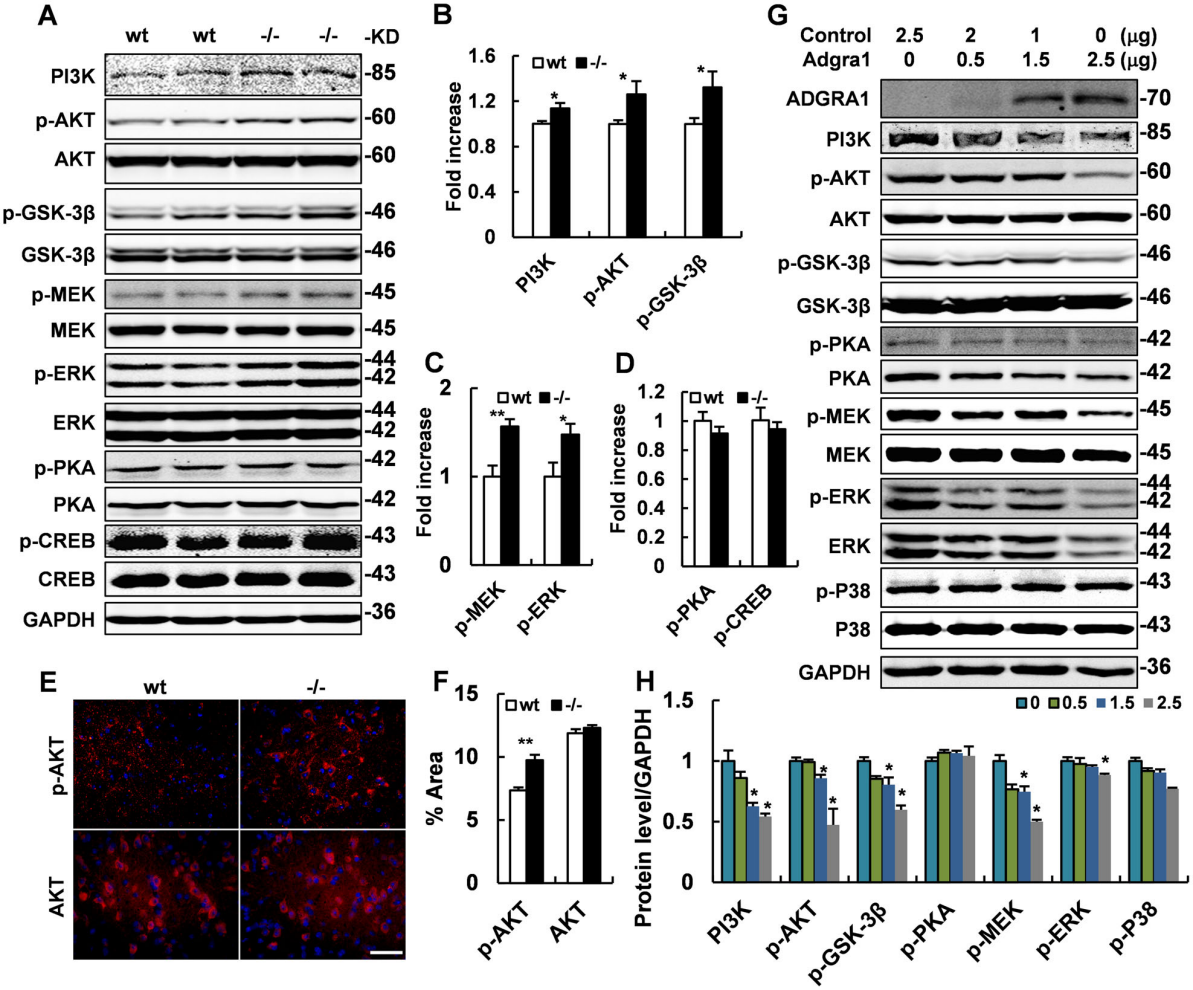
ADGRA1 deficiency in male mice enhances PI3K/AKT/GSK3β and MEK/ERK pathways in the hypothalamus. **A** Protein levels of GPCR mediated pathways analyzed by western blot. GAPDH is used as a loading control. **B** Quantitative analysis of relative intensities of PI3K/AKT/GSK3β pathway. Relative intensities of p-AKT and p-GSK3β normalized to total AKT and GSK3β, respectively, *n* = 5/group. **C** Quantitative analysis of relative intensities of MEK/ERK pathway. Relative intensities of p-MEK and p-ERK normalized to total MEK and ERK, respectively, *n* = 5/group. **D** Quantitative analysis of relative intensities of PKA/CREB pathway. Relative intensities of p-PKA and p-CREB normalized to total PKA and CREB, respectively, *n* = 5/group. **E** Representative images of IF of phosphorylated and total AKT in the hypothalamus of both genotypes. Scale bar, 50 μm. **F** Quantitative analysis of positive areas of p-AKT and total AKT in the images, *n* = 5/group. **G** pcDNA3.1b (−) and pcDNA3.1-*Adgra1* vectors are transfected into Neuro2A cells and the cell lysates from transfected cells are assessed by western blot. **H** Quantitative analysis of relative intensities of pathways. Relative intensities of phosphorylated proteins normalized to total proteins, respectively, *n* = 3 independent experiments. *p < 0.05, **p < 0.01 vs. controls. Data represent mean ± S.E.M.

To clarify how ADGRA1 affects the metabolic process, we analyzed the classical pathways downstream of GPCRs in hypothalamus and found the PI3K/AKT pathway was activated in *Adgra1*^*−/−*^ male mice by western blotting (Fig. 7A) and IF staining (Fig. 7E). As a result, activated AKT facilitated the phosphorylation of GSK3β (Fig. 7A). In addition, MEK/ERK was also upregulated while PKA/CREB was unaffected in the absence of ADGRA1 in male mice (Fig. 7A). All these differences were verified by quantitative analysis (Fig. 7B–D, F). Next, we detected these pathways in transfected Neuro2A cells, in which the ADGRA1 was over-expressed. Results showed both PI3K/AKT/GSK3β and MEK/ERK pathways were suppressed in a dose-dependent association with the expression of ADGRA1, while the other pathways were unchanged (Fig. 7G, H). The signaling analyses were also conducted in female mice and the results showed no significant differences (Supplementary Fig. 11h). To explore the possible molecular mechanism responsible for the differences between male and female mice, we tested *Adgra1* mRNA level in wt mice and found the expression of *Adgra1* in male mice was higher than that in female mice (Supplementary Fig. 11i). While the *Adgra1* mRNA in natural Neuro2A cells was increased under the stimulation of testosterone with a selected range of concentrations (Supplementary Fig. 11j).

### Testosterone treatment has a slight effect on the metabolic status in female mice

Consequently, we performed testosterone treatment in female mice and results showed that female mice of two genotypes exhibited similar metabolic rate at basal condition. However, after treated with testosterone for a month, *Adgra1*^*−/−*^ female mice displayed an increasing trend in metabolic rate albeit no significant differences (*p* < 0.07) when compared with controls (Supplementary Fig. 12a–i). Body weights and core temperature showed no significant differences (Supplementary Fig. 13a, b). Serum detection proved the treatment effectively improved the testosterone levels in females (Supplementary Fig. 13c). Moreover, testosterone treatment in wt female mice also induced increased *Adgra1* mRNA in brain (Supplementary Fig. 13d). Androgen receptor (*Ar*) mRNAs were unaffected both in mice models and transfected cells (Supplementary Fig. 13e, f).

## Discussion

Metabolism homeostasis is essential for the body growth and development. Imbalance of energy metabolism leads to several metabolic diseases. Typically, the excessive intake of energy contributes to the development of obesity, which is a rapidly growing health concern for the modern society^14,15^, while hypermetabolism, characterized by the increased energy expenditure and weight loss, is a manifestation of hyperthyroidism^16^. The pathogenesis of these metabolic diseases and the molecular mechanisms underlying the energy balance are only partially understood. A well-established view is that hypothalamus is a metabolic center which integrates information from other brain regions and provides a coordinated response by arousing specific metabolism performance, including food intake, glucolipid metabolism, insulin sensitivity, BAT thermogenesis, and physical activities^17,18^. Many neuropeptides in hypothalamus, such as proopiomelanocortin, orexin, neuropeptide Y, agouti-related peptide, melanin-concentrating hormone, TRH, and CRH have been proved to be vital modulators of metabolism homeostasis^19,20^. In line with these findings, the high expression of *Adgra1* in hypothalamus together with the alterations of hypothalamic pathways suggest that hypothalamus may be the controlling center in the metabolic process of *Adgra1*^*−/−*^ male mice.

Excessive energy expenditure is the cause of thinness in *Adgra1*^*−/−*^ male mice, where the energy goes is a point of concern. Total energy expenditure is divided into three main components: obligatory energy expenditure, locomotive energy expenditure, and adaptive thermogenesis-mediated energy expenditure^21^. BAT is a mainly thermogenic tissue in rodents and has been reported to be heavily innervated by sympathetic nerves from hypothalamus in the adaptive thermogenesis. For instance, inhibition of hypothalamic AMPK enhances thermogenesis in adipocytes through activating the SNS^22^. Activated leptin signaling in hypothalamus controls the energy balance by regulating SNS outflow to BAT^23^. Consistently, *Adgra1* deficiency in hypothalamus regulates BAT thermogenesis through activating the SNS evidenced by the upregulated TH and β3-AR expression levels. TH is the rate-limiting enzyme in the synthesis of the sympathetic neurotransmitter, norepinephrine (NE), and β3-AR is the main receptor for NE in adipocytes. In this process, sympathetic premotor neurons in hypothalamus are activated by external stimulation, then the NE is synthesized and released in response to coordinated nerve impulses, binding to the β3-AR on the adipose tissues^24,25^. In BAT, the interaction activating the PGC1, which co-activates members of the peroxisome proliferator-activated receptor family, thus UCP1 is expressed, as a result, the thermogenesis is enhanced^21^. In WAT, HSL, a critical enzyme in lipid hydrolysis, is activated by adrenergic receptors and consumes fuel for adaptive thermogenesis^26^. All these proteins involved in thermogenic process are activated in *Adgra1*^*−/−*^ male mice, confirming that these mice devote excessive energy expenditure to thermogenesis via SNS.

**Fig. 8:**
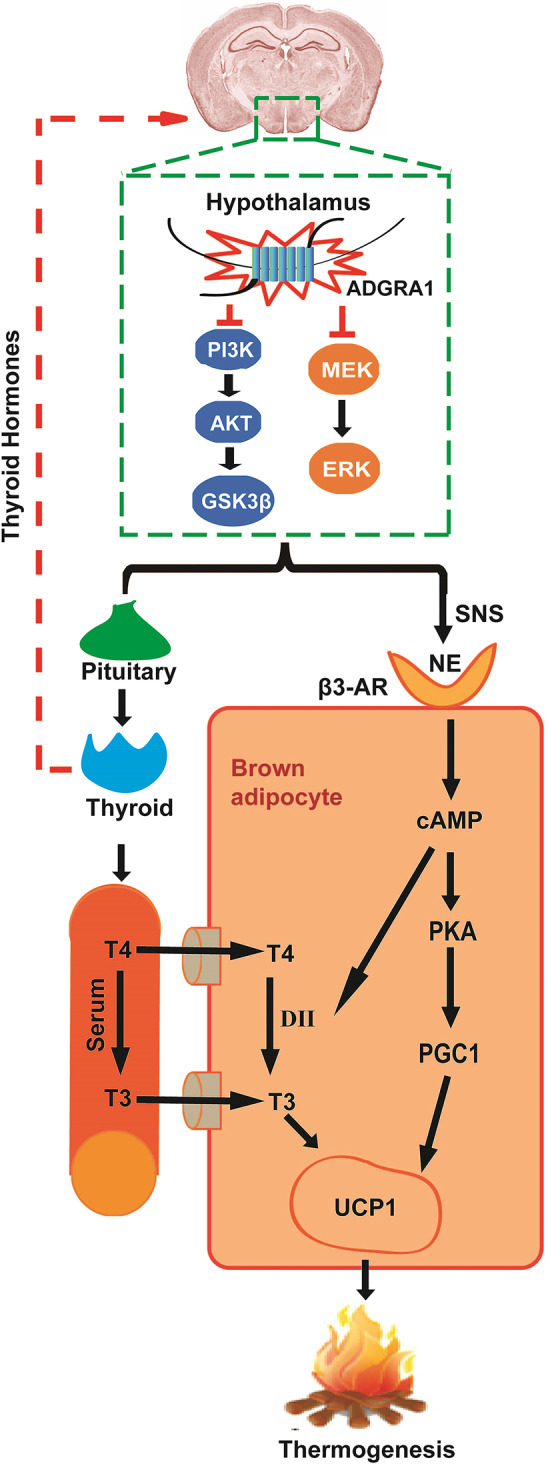
Schematic overview summarizing the physiological effects of ADGRA1 on energy balance in male mice. ADGRA1 in male mice modulates the PI3K/AKT/GSK3β and MEK/ERK pathways in the hypothalamus, negatively regulating both hypothalamus–pituitary–thyroid axis and SNS. NE is released from SNS and interacts with β3-AR in brown adipocytes. On one hand, the interaction initiates a cascade of reaction activating the PGC1 and resulting in the expression of UCP1. On the other hand, the interaction can also activate the DII, which promotes the conversion of T4 to T3, as a result, the UCP1 is expressed. Together, heat is generated.

On the other hand, the hypothalamus can also affect energy expenditure and thermogenic process by means of hypothalamic–pituitary–thyroid axis. Our results illustrate that the activated hypothalamic–pituitary–thyroid axis in *Adgra1*^*−/−*^ male mice results in excessive production and release of THs. The THs traveling in the blood, on one hand, re-enter CNS and feedback on hypothalamus, stimulating neural signals or regulating TRH production; on the other hand, directly act on thermogenic organs. The central effects of THs on energy metabolism via adipose tissues are evidenced by the reports that either hyperthyroidism or central administration of T3 can inhibit the activation of hypothalamic AMPK and facilitate BAT thermogenesis by increased SNS activity^27^. Therefore, it is reasonable to speculate that the excessive THs in *Adgra1*^*−/−*^ male mice may re-act on hypothalamus and activate SNS, facilitating thermogenesis in adipose tissues. In addition, activated SNS initiates a cascade of reactions to upregulate type II thyroxine deiodinase (DII), which promotes the conversion of T4 to T3. T3 is a ligand for thyroid hormone receptors, which are assembled on the UCP1 enhancer in BAT, thus the expression of UCP1 is increased^21^. Together, these findings implicate that the increased energy expenditure and thermogenesis in the deficiency of *Adgra1* are integrated reactions responded by both hormones and neural signals and the underlying process might be complex and multi-layered (Fig. 8).

The crucial roles of neural and endocrine systems in controlling energy homeostasis have been largely studied, in which GPCR signaling pathways are increasingly discovered to be key regulators^28–30^. PI3K/AKT and ERK in hypothalamus have been proved to regulate energy balance by stimulating SNS activity to adipocytes^31,32^. Similarly, our results show that activated PI3K/AKT/GSK3β and MEK/ERK pathways are responsible for the abnormal metabolic process in male mutant mice. GSK3β has been reported to promote dendrite formation by regulating the activity of the key dendrite formation effector in sympathetic neurons^33^, implying that PI3K/AKT/GSK3β may regulate the development of the hypothalamic sympathetic neurons. MEK/ERK in hypothalamus may participate in the survival of sympathetic neurons in consideration of its important function in cell proliferation and apoptosis of biological processes. Furthermore, both in vivo and in vitro studies suggest that *Adgra1* transmits suppressive signals to its downstream pathway. The other research has shown that the deletion of GPR17 inhibits the Gαi protein and activates cAMP-PKA pathway, promoting energy expenditure and reducing body weight^34^. However, the PKA/CREB pathway is unchanged in the *Adgra1*^*−/−*^ male mice, implying the unaffected Gαi levels. Nevertheless, the dissociation of the heterotrimer permits the free Gβγ to interact with the downstream effectors independent of Gαi. A classical paradigm of Gi signaling is that Gβγ subunits trigger the generation of phosphatidylinositol (3,4,5)-triphosphate (PIP3), as a result, the PI3K is activated^35–38^. Accordingly, it is plausible that Gβγ-subunit from Gi may function on the *Adgra1* and initiate the downstream signals in our study. Further studies are needed to clarify how effectively the ADGRA1 couples with Gi protein and to define whether the targets of Gi signaling can be harnessed in disease models.

In addition to the physiological function of *Adgra1*, another notable discovery in the current study is that abnormal metabolic phenotype is only occurred in *Adgra1*^*−/−*^ male mice but not in females. It is obvious that alterations of pathways in transfected cells are dose-dependent on the expression of ADGRA1 and mRNA of *Adgra1* is significantly higher in males than that in females, suggesting that the expression of *Adgra1* may contribute to the discrepancy of metabolic phenotypes. Presumably, deletion of *Adgra1* in male mice may lead to more remarkable changes in metabolism, while the lack of *Adgra1* in female mice is not sufficient to cause the corresponding alterations. In addition, such sex-specific features are often associated with gonadal hormones or their corresponding receptors, which may affect the energy metabolism through regulating output of hypothalamic neurotransmitter^39–42^. Consistent with these studies, we find that testosterone promotes *Adgra1* mRNA level in a selected range of doses in Neuro2A cells. Deeply, *Adgra1*^*−/−*^ female mice treated with testosterone exhibited slightly higher metabolic rate. Furthermore, testosterone treatment in female wt mice upregulated their *Adgra1* mRNA levels, raising the possibility that testosterone-induced expression pattern of *Adgra1* was one of the explanations for the sex-specific metabolism. However, the absence of significant difference in metabolism between *Adgra1*^*−/−*^ female mice and their littermate controls after testosterone treatment indicated that testosterone was not the only functional element for the gender specificity on metabolic changes. The unaffected testosterone-AR signaling in the *Adgra1* deficiency mouse model suggested that testosterone could exert a direct effect on *Adgra1* gene expression in the brain. Accumulating evidence implicated that the difference in *Adgra1* expression levels is associated with testosterone level and the sex-specific metabolism may be dependent on expression level of *Adgra1* between genders. However, the molecular mechanisms should be further investigated.

In conclusion, *Adgra1* deletion leads to decreased body weight and increased energy expenditure in male but not in female mice. *Adgra1* in male mice not only modulates the hypothalamus–pituitary–thyroid axis but also controls the outflow of sympathetic nerve to adipocytes, which synergistically mediates thermogenesis in adipose tissues. The PI3K/AKT/GSK3β and MEK/ERK pathways initiated by deficiency of ADGRA1 in the hypothalamus regulate whole-body metabolic homeostasis. These findings provide insights into the physiological function of *Adgra1* for the first time and support *Adgra1* as a new negative regulator in energy homeostasis. Modulation of *Adgra1* signaling may uncover new therapeutic strategies to control thermogenesis and combat metabolic disorders.

## Materials and methods

### Mice

*Adgra1*^*−/−*^ mice were generated by a standard cre-loxp strategy as described in Supplementary Fig. 1. Mice were housed in groups of 3–5 on a 12-h light/dark (7 AM/7 PM) cycle under conditions of controlled temperature (23 °C) and humidity with ad libitum access to standard laboratory chow food and water. Food was only withdrawn if required for an experiment. Experiments were performed in both genders.

### Body composition

Body composition (fat and lean mass) was assessed with the EchoMRI whole-body composition analyzer by quantitative nuclear magnetic resonance relaxometry.

### Glucose tolerance test (GTT) and insulin tolerance test (ITT)

Overnight-fasted mice were given glucose, the dosage was chosen based on preliminary body weight (2 mg/g body weight, intraperitoneal, ip). For ITT, mice were fasted for 8 h and were given insulin (0.75 mIU/g, ip). Tail blood was collected, blood glucose was monitored at indicated time points with a handheld glucometer (Roche) after injection.

### Heart rate and blood pressure

Heart rate and blood pressure of wt and mutant mice were monitored with a BP-98A Specimen platform (Softron).

### Body temperature and cold exposure

Core body temperature was measured using a rectal probe thermometry connected to a digital thermometer inserted 1-cm deep at controlled room temperature (23 °C). Mice performed in cold exposure test were caged individually for 4 h in a room with steady temperature of 6 °C and provided with food and water ad libitum. Body temperature was monitored hourly.

### Metabolic analyses

Mice were individually housed and acclimatized for 48 h in the metabolic cages with ad libitum access to food and water. Next, oxygen consumption (VO_2_), carbon dioxide production (VCO_2_), total and ambulating locomotor activities were collected continuously. Afterward, mice fasted for 24 h and the metabolic parameters were collected as above. Energy expenditure (EE) and respiratory exchange ratio (RER) were determined by the following equations: EE = (3.815 + 1.232*RER)*VO_2_, RER = VCO_2_/VO_2_. The results were all normalized to body weight.

### mRNA expression analyses

Total RNA was isolated from varieties of mouse tissues using the Trizol method (Invitrogen, Carlsbad, CA) according to the manufacturer’s instructions. Real-time PCR was performed to evaluate mRNA levels using the SYBR Premix Ex Taq kit (Takara, Dalian, China) on an Eppendorf Mastercycler system. Samples in this study were assessed in triplicate, and the results were normalized to the *β*-actin. The primers for PCR assays were listed in Supplementary Table 1.

### Western blotting

Protein was extracted from cells and tissues using RIPA lysis buffer with protease and phosphatase inhibitor cocktail (Roche). Proteins in equal amounts were separated by SDS-PAGE, then transferred to nitrocellulose membranes (GE). The nitrocellulose membranes were incubated overnight with specific primary antibodies at 4 °C and were probed with fluorescent secondary antibodies at room temperature for 1–2 h. Finally, western blot images were obtained by Odyssey infrared fluorescence imaging system (Li-COR). GAPDH was used as the protein loading control. All antibodies were listed in Supplementary Table 2. Quantitative analysis of band intensity of each protein was performed using Image J software.

### Biochemical assays

Blood samples were collected from male mice at the age of 23 weeks, female mice without testosterone treated at the age of 17 weeks, and female mice with testosterone treated at the age of 16 weeks. The serum was analyzed using an automatic biochemical analyzer. Insulin and corticosterone levels were measured by insulin ELISA kit (Mercodia, Sweden) and corticosterone ELISA kit (DRG, Germany), respectively. Thyroid hormones (THs), prolactin (PRL), and testosterone levels were measured by radioimmunoassay (RIA).

### Histological and immunofluorescence (IF) analyses

Tissues including brains, BAT, and WAT were dissected and fixed. For hematoxylin and eosin (HE) staining, it was performed routinely to administrate the histology analysis and the lipid droplet-positive areas of adipocytes were quantified using Image J software. For IF, the brain sections were deparaffinized and antigen unmasked, blocked, incubated with primary antibodies at 4 °C overnight, and incubated with fluor-conjugated secondary antibodies at room temperature for 2 h. Nuclei were visualized with 4′,6-diamidino-2-phenylindole (DAPI) (Invitrogen) staining. Slides were observed by the fluorescence microscope (Nikon Eclipse 90i). Antibodies were listed in Supplementary Table 2. The fluorescence intensity of each section from mouse was quantified using Image J software.

### Cell culture and transfection

The Neuro2A cell line was a gift from the laboratory of Shanghai Engineering and Technology Research Center for Model Animals and was routinely cultured in standard DMEM medium (Hyclone) supplemented with 10% (vol/vol) FBS (Gibco) within a humidified incubator containing 5% CO_2_ at 37 °C. *Adgra1* cDNA was inserted into the pcDNA3.1b (−) and pEGFP-N2 (Invitrogen) vectors. Vectors with correct splicing were transfected into Neuro2A cells with Lipofectamine 3000 transfection reagent (Invitrogen). After 48 h, cells were collected for IF and western blot analyses. In the testosterone treatment, the natural Neuro2A cells were plated in a 6-well dish at a density of 5 × 10^5^ cells/well and the medium was supplemented with different concentrations of testosterone (0, 10, 20, 50, 100, and 200 ng/ml). For the transfected cells, the medium was supplemented with PBS and testosterone (50 ng/ml), respectively.

### Statistical analysis

Data were presented as means ± standard error (S.E.M). Comparison between two independent data sets was determined by a two-tailed Student’s *t*-test. One-way and two-way repeated-measures ANOVA analysis by GraphPad Prism Software Version 8 were used for multiple comparisons. Post hoc statistics were conducted using Sidak’s multiple comparison test. *p* < 0.05 was considered to define statistical significance. Although no statistical analysis was performed to determine effect sizes aforehand, sample sizes here were similar to those researched in the same type of studies^34,43,44^.

## Supplementary information

Supplementary Figure Legends

Supplementary Figure 1

Supplementary Figure 2

Supplementary Figure 3

Supplementary Figure 4

Supplementary Figure 5

Supplementary Figure 6

Supplementary Figure 7

Supplementary Figure 8

Supplementary Figure 9

Supplementary Figure 10

Supplementary Figure 11

Supplementary Figure 12

Supplementary Figure 13

Supplementary table 1

Supplementary table 2
