# Supplementary material for: ADGRA1 negatively regulates energy expenditure and thermogenesis through both sympathetic nervous system and hypothalamus–pituitary–thyroid axis in male mice
Source: Cell Death Dis. 2021 Apr 6;12(4):362. doi: 10.1038/s41419-021-03634-7 (PMC8024368; doi:10.1038/s41419-021-03634-7)
Supplement: Supplementary file 1 — Supplementary Figure Legends [file 41419_2021_3634_MOESM1_ESM.docx]

**Supplementary Figure Legends:**

**Supplementary Fig. 1 Targeted deletion of *Adgra1* in mice.** **a** The graph of *Adgra1* targeting strategy. Black boxes refer to the coding regions of exons. The targeting vector contains 1729 bp of 5′ and 3973 bp of 3′ homologous fragments. PGK-Neo cassette attached with loxP sequence is inserted into intron 4, the HSV-TK is used for negative selection. The other loxP sequence is inserted into intron 3. Mice with loxP-flanked alleles of exon 4 are bred to cre transgenic mice to remove the exon 4, inducing global deletion of *Adgra1* in the mice. The bold arrows on the allele represent the inserted loxP sequences and the filled black ellipses refer to the frt elements. P1-P6, the primers and their relative positions for genotyping are marked as arrows. **b** PCR analysis of ES cell clones is conducted using the primers P1 and P2 for the 5′ homologous arms and P3 and P4 for the 3′ homologous arms. Products are verified by DNA sequencing. **c** Routine genotyping of wt, *Adgra1^+/-^*, and *Adgra1*^-/-^ mice are identified by PCR using primers P5-P6 on mouse tail genomic DNA. **d** Reverse-transcription PCR is performed for the expression of *Adgra1* mRNA using RNAs prepared from brains of three different genotypes with specific primers of *Adgra1*. *Gapdh* is used as a loading control. **e** Western blot analysis of ADGRA1 protein in the brain tissues from wt and *Adgra1*^-/-^ mice. GAPDH is used as a loading control. **f** Expression pattern of ADGRA1 protein in hypothalamus, cortex, amygdala and hippocampus of wt and knockout adult mouse revealed by IF. Scale bar, 50 μm.

**Supplementary Fig. 2 Comparable body weight in *Adgra1^-/-^* female mice and their littermate controls. a** Body weight curves of *Adgra1^-/-^* female and control mice, n=7-8/group. **b** Body mass (total, fat and lean weight) of *Adgra1^-/-^* female and control mice, n=6-8/group. **c** Serum chemistries in female mice, n=5/group. **d** Representative images of HE stained BAT and WAT from *Adgra1^-/-^* female and control mice. Scale bar, 50 μm, n=5/group. **e** Basal blood glucose level after fasted overnight of *Adgra1^-/-^* female and wt mice, n=7-8/group. **f** GTT in *Adgra1^-/-^* female and wt mice, n=7-8/group. BG, blood glucose.

**Supplementary Fig. 3 Morphological analysis of brain in *Adgra1^-/-^* male mice and their littermate controls.** **a** Representative images of brains of *Adgra1^-/-^* male mice and their littermate controls. **b** Brain weights of *Adgra1^-/-^* male mice and their littermate controls, n=6/group. **c** Rate of brain weights to their body weights, n=6/group. **d** Representative images of HE stained brain from *Adgra1^-/-^* male mice and controls. Scale bar, 50 μm. Data represent mean ± S.E.M.

**Supplementary Fig. 4 Analysis of organ function in *Adgra1^-/-^* male mice and their littermate controls.** **a** Representative images of the organs of *Adgra1^-/-^* male mice and their littermate controls. **b** Organ weights of *Adgra1^-/-^* male mice and their littermate controls, n=6/group. **c** Rate of organ weights to their body weights, n=6/group. **d** Serum chemistries in male mice for liver function, n=6/group. A/G, albumin/globulin; GLOB globulin; ALB, albumin; ALP, alkaline phosphatase; ALT, alanine transaminase; AST aspertate aminotransferase; CHE, cholinesterase; D-BIL, direct bilirubin; GGT, gamma-glutamyl transferase; T-BIL, total bilirubin; TP, total protein. **e** Serum chemistries in male mice for cardiovascular function, n=6/group. Hcy-s, homocysteine; CK, creatine kinase; LDH, lactate dehydrogenase. **f** Serum chemistries in male mice for kidney function, n=6/group. BUN, blood urea nitrogen; CRE, creatinine; UA, uric acid. Data represent mean ± S.E.M.

**Supplementary Fig. 5 ADGRA1 is predominantly expressed in the CNS. a-b** Expression profiles of *Adgra1* mRNA in wt adult mice (a) and human (b) assessed by real-time PCR. *β-actin* is examined as an internal control. Data represent mean ± S.E.M. **c** Representative images of IF of ADGRA1 with NEUN, a neuron marker, and GFAP, a astrocytes marker, separately on sections of cortex from wt mice. Scale bar, 50 μm. **d** Representative images of IF of EGFP protein and ADGRA1-EGFP fusion protein in transfected Neuro2A cells. Scale bar, 20 μm.

**Supplementary Fig. 6** **NEUN staining on brain sections of *Adgra1^-/-^* male mice and their littermate controls.** Representative images of IF of NEUN on sections of hypothalamus (a), amygdala (b), hippocampus (c), and cortex (d) from *Adgra1^-/-^* male mice and their littermate controls. Scale bar, 50 μm.

**Supplementary Fig. 7 GFAP staining on brain sections of *Adgra1^-/-^* male mice and their littermate controls.** Representative images of IF of GFAP on sections of hypothalamus (a), amygdala (b), hippocampus (c), and cortex (d) from *Adgra1^-/-^* male mice and their littermate controls. Scale bar, 50 μm.

**Supplementary Fig. 8 IBA1 staining on brain sections of *Adgra1^-/-^* male mice and their littermate controls.** Representative images of IF of IBA1, a microglial marker on sections of hypothalamus (a), amygdala (b), hippocampus (c), and cortex (d) from *Adgra1^-/-^* male mice and their littermate controls. Scale bar, 50 μm.

**Supplementary Fig. 9 OLIG2 staining on brain sections of *Adgra1^-/-^* male mice and their littermate controls.** Representative images of IF of OLIG2, an oligodendroglia marker on sections of hypothalamus (a), amygdala (b), hippocampus (c), and cortex (d) from *Adgra1^-/-^* male mice and their littermate controls. Scale bar, 50 μm.

**Supplementary Fig. 10 Comparable metabolic rate in *Adgra1^-/-^* female mice and their littermate controls. a-g** At the age of 17 weeks, the metabolic cage studies assess O_2_ consumption in curve diagram (a) and histogram (b), CO_2_ production (c), energy expenditure (d), RER (e), locomotor activities (f), and food intake (g) during light and dark of *Adgra1^-/-^* female and control mice, n=4-6/group. EE, energy expenditure. **h-i** O_2_ consumption for carbonhydrate (h) and fat (i) during light and dark of *Adgra1^-/-^* female and control mice, n=4-6/group. Data represent mean ± S.E.M.

**Supplementary Fig.11 Adaptive thermogenesis in *Adgra1^-/-^* female mice is unaffected. a** Rectal temperature of *Adgra1^-/-^* female and control mice, n=4-7/group. **b** Core temperature of *Adgra1^-/-^* female and control mice when they are exposed to cold (6℃) for 4 h, n=4-7/group. **c** Heart rate in female mice of both genotypes, n=7-8/group. **d** Blood pressure in female mice of both genotypes, n=7-8/group. SBP, systolic blood pressure. DBP, diastolic blood pressure. MBP, mean blood pressure. **e** mRNA levels of thermogenic genes in BAT assessed by real-time PCR, n=5/group. **f** mRNA levels of *Th* and *Adrb3* in BAT and WAT assessed by real-time PCR, n=5/group. **g** Protein levels in BAT and WAT of *Adgra1^-/-^* female and control mice analyzed by western blot. GAPDH is used as a loading control. **h** Protein levels of AKT/GSK-3β pathway in the hypothalamus of female mice analyzed by western blot. GAPDH is used as a loading control. **i** mRNA levels of *Adgra1* in brain regions of wt male and female mice, n=5/group. **j** mRNA levels of *Adgra1* in Neuro2A cells after stimulated by testosterone, n=3 independent experiments. *p < 0.05, **p < 0.01, ***p < 0.001 vs. controls. Data represent mean ± S.EM.

**Supplementary Fig. 12 Testosterone treatment has s slight effect on the metabolic status in female mice*.* a** At the age of 11 weeks, metabolic cage studies were conducted on the female mice to assess O_2_ consumption in curve diagram, n=6/group. **b** After testosterone treatment for a month, metabolic cage studies were conducted on the same female mice at the age of 16 weeks to assess O_2_ consumption in curve diagram, n=6/group. **c** O_2_ consumption in histogram for the female mice before and after testosterone treated, n=6/group. **d** CO_2_ production for the female mice before and after testosterone treated, n=6/group. **e** energy expenditure for the female mice before and after testosterone treated, n=6/group. **f** RER for the female mice before and after testosterone treated, n=6/group. EE, energy expenditure. **g-h** O_2_ consumption for carbonhydrate (g) and fat (h) for the female mice before and after testosterone treated, n=6/group. **i** Locomotor activities for the female mice before and after testosterone treated, n=6/group. “-” represents female mice without testosterone treatment; “+” represents female mice with testosterone treatment. **^#^**p < 0.07, *p < 0.05 vs. controls. Data represent mean ± S.E.M.

**Supplementary Fig. 13 Testosterone treatment upregulates *Adgra1* mRNA level in female mice. a** Body weight curves of *Adgra1^-/-^* female and control mice, at the age of 12 weeks, testosterone was injected intraperitoneally into mice every day for consecutive 30 days (3mg/kg body weight), n=6/group. **b** Serum testosterone levels for the female mice with and without testosterone treated at the age of 16-17 weeks, n=5-6/group. **c** Core temperature of female mice before and after testosterone treated, n=6/group. **d** *Adgra1* mRNA level in wt female mice before and after testosterone treated, n=5-6/group. **e** *Ar* mRNA level in *Adgra1^-/-^* female and control mice before and after testosterone treated, n=5-6/group. **f** *Ar* mRNA level in transfected cells with and without testosterone treated, n=3 independent experiments. “Control” represents that cells were transfected with pcDNA3.1b (-) vector, “Adgra1” represents that cells were transfected with pcDNA3.1-*Adgra1* vector. T, testosterone. “-” represents female mice without testosterone treatment; “+” represents female mice with testosterone treatment. *p < 0.05 vs. controls. Data represent mean ± S.E.M.
