# Supplementary material for: ADGRA1 negatively regulates energy expenditure and thermogenesis through both sympathetic nervous system and hypothalamus–pituitary–thyroid axis in male mice
Source: Cell Death Dis. 2021 Apr 6;12(4):362. doi: 10.1038/s41419-021-03634-7 (PMC8024368; doi:10.1038/s41419-021-03634-7)
Supplement: Supplementary file 15 — Supplementary table 1 [file 41419_2021_3634_MOESM15_ESM.docx]

| **Supplementary Table 1: Primers used in the current study.** | | |  | |  |
| --- | --- | --- | --- | --- | --- |
| Primer | Sequence | Primer | | Sequence | |
| P1 | 5′- CTCCCGCGAGGCTTTGCTCTATGAAT-3′ | Th forward | | 5′-AAGGGCCTCTATGCTACCCA-3′ | |
| P2 | 5′-CTGAGCCCAGAAAGCGAAGGA-3′ | Th reverse | | 5′-GCCAGTCCGTTCCTTCAAGA-3′ | |
| P3 | 5′- GTGCCACTCCCACTGTCCTTTCC-3′ | Adrb3 forward | | 5′-ACGCCGAGACTACAGACCATAACC-3′ | |
| P4 | 5′- GGTGAGGTAGGGGAGCTGTCGTGATG-3′ | Adrb3 reverse | | 5′-GACGAAGAGCATCACAAGGAGGG-3′ | |
| P5 | 5′-TTTGAACTGTGACCTCCAGCCCTTTA-3′ | Crh forward | | 5′-GGATCTCACCTTCCACCTTCTGCG-3′ | |
| P6 | 5′-TAAGAAGTCCCAGTGAGGGCTAAGA-3′ | Crh reverse | | 5′-TTCCCGATAATCTCCATCAGTTTCCT-3′ | |
| Adgra1 forward | 5′-AGGGGAGTTCCTGCATCCT-3′ | Trh forward | | 5′-TGATGATGGCTCTGGCTTTGA-3′ | |
| Adgra1 reverse | 5′-GGCCTTTCCGGCTTATCCG-3′ | Trh reverse | | 5′-CTGGCCGGACCTGGACTTT-3′ | |
| Gapdh forward | 5′-CCTCGTCCCGTAGACAAAATGGT-3′ | Gh forward | | 5′-CGAGCGTGCCTACATTCCC-3′ | |
| Gapdh reverse | 5′-TTGAGGTCAATGAAGGGGTCGT-3′ | Gh reverse | | 5′-CGAGGTGCCGAACATCAGG-3′ | |
| β-actin forward | 5′-CCTAAGGCCAACCGTGAAA-3′ | Prl forward | | 5′-GGAACAAGCCCTGAAAGTC-3′ | |
| β-actin reverse | 5′-AGAGCATAGCCCTCGTAGAT-3′ | Prl reverse | | 5′-CCAAACAAAGTAGATCCCATT-3′ | |
| Pgc1a forward | 5′-TGAGAACAAGACTATTGAGCGAACC-3′ | Pomc forward | | 5′-CTGTTGCTGGCCCTCCTGCTT-3′ | |
| Pgc1a reverse | 5′-GGAGTGGCTGCCTTGGGTA-3′ | Pomc reverse | | 5′-CCTCCGCCGACTCGTTCTCA-3′ | |
| Cox5b forward | 5′-GCAGGCTACTGGGCTGGAGA-3′ | Lhb forward | | 5′-GCATCACCTTCACCACCAGCAT-3′ | |
| Cox5b reverse | 5′-TTGTTGCTGATGGACGGGAC-3′ | Lhb reverse | | 5′-GGTCACAGGCCATTGGTTGAGT-3′ | |
| Cox7a1 forward | 5′-AGGCTCTGGTCCGGTCTTT-3′ | Fshb forward | | 5′-GCATCTTATTCTGGTGCTGG-3′ | |
| Cox7a1 reverse | 5′-TGTACTGGGAGGTCATTGTCG-3′ | Fshb reverse | | 5′-GGTACATACTTTCTGGGTATTGG-3′ | |
| Cidea forward | 5′-CCTGGTTACGCTGGTGCTG-3′ | Tshb forward | | 5′-ATTTCTCCTTCCCTGTCGC-3′ | |
| Cidea reverse | 5′-GCTATTCCCGATTTCTTTGGTT-3′ | Tshb reverse | | 5′-GGCTTGGTGCAGTAGTTGG-3′ | |
| Cycs forward | 5′-AAATCTCCACGGTCTGTTCG-3′ | Tshr forward | | 5′-CTTACATGACTTCGGTCCCT-3′ | |
| Cycs reverse | 5′-TGCCCTTTCTCCCTTCTTC-3′ | Tshr reverse | | 5′-CTAGATTTGTGCCTGGTGG-3′ | |
| Tfam forward | 5′-CCCTCGTCTATCAGTCTTGTC-3′ | Atp1b1 forward | | 5′-ACCGAAGCCTCCCAAGAAT-3′ | |
| Tfam reverse | 5′-CTCCTTCTCCATACCCATCA-3′ | Atp1b1 reverse | | 5′-GTATCCGCCCATCCCAAAG-3′ | |
| Pparg forward | 5′-AGACCACTCGCATTCCTTT-3′ | Dio1 forward | | 5′-CCCCTGGTGTTGAACTTTGG-3′ | |
| Pparg reverse | 5′-CCCACAGACTCGGCACTCA-3′ | Dio1 reverse | | 5′-GCAGATCCTGCCCTCCTGT-3′ | |
| Adipoq forward | 5′-CTCTTAATCCTGCCCAGTCAT-3′ | Tg forward | | 5′-AGACATTCTCAGCGGACACC-3′ | |
| Adipoq reverse | 5′-TCCAACCTGCACAAGTTCC-3′ | Tg reverse | | 5′-TCCTTCAGGGCATTTCACA-3′ | |
| Fabp4 forward | 5′-TTTGTGGGAACCTGGAAGC-3′ | Ucp1 forward | | 5′-GGCAGCCTACAGAGGTCGTG-3′ | |
| Fabp4 reverse | 5′-CCTGTCGTCTGCGGTGATT-3′ | Ucp1 reverse | | 5′-CGTCGGTCCTTCCTTGGTG-3′ | |
| Egfp-Adgra1 forward | 5′-TCGAGCTCAAGCTTCGAATTCAGAC  TACCCAGGAAAACTGAGTCG-3′ | pCDNA3.1-Adgra1  forward | | 5′-AACGGGCCCTCTAGACTCGAGAGAC  TACCCAGGAAAACTGAGTCG-3′ | |
| Egfp-Adgra1 reverse | 5′-TGGCGACCGGCCGGTGGATCCGC  ACTGTAGTTTCATTTTTCCAGG-3′ | pCDNA3.1-Adgra1  reverse | | 5′-CTTGGTACCGAGCTCGGATCCCGC  ACTGTAGTTTCATTTTTCCA-3′ | |
| Ar forward | AGTTAGGGCTGGGAAGGGTC | Ar reverse | | GCCGGGAGGTGCTATGTTAG | |
