# Supplementary material for: ADGRA1 negatively regulates energy expenditure and thermogenesis through both sympathetic nervous system and hypothalamus–pituitary–thyroid axis in male mice
Source: Cell Death Dis. 2021 Apr 6;12(4):362. doi: 10.1038/s41419-021-03634-7 (PMC8024368; doi:10.1038/s41419-021-03634-7)
Supplement: Supplementary file 16 — Supplementary table 2 [file 41419_2021_3634_MOESM16_ESM.docx]

| **Supplementary Table 2: Antibodies used in Western blotting and IF assays.** | | | |
| --- | --- | --- | --- |
| Antibodies | Source | Cat.No. |  |
|  |  |  |  |
| ADGRA1 Rabbit pAb | Abcam | ab122814 |  |
| NeuN Mouse mAb | Millipore | MAB377 |  |
| GFAP Mouse mAb | Millipore | MAB3402 |  |
| β3-AR Rabbit pAb | Abcam | ab94506 |  |
| IBA1 Rabbit pAb | Abcam | ab178846 |  |
| OLIG2 Rabbit pAb | Abcam | ab109186 |  |
| UCP1 (4E5) Mouse mAb | Santa cruz | sc293418 |  |
| PGC1-α Rabbit pAb | Santa cruz | sc13067 |  |
| TH Mouse mAb | Immunostar | 22941 |  |
| Phospho-HSL (Ser660) Rabbit pAb | Cell Signaling Technology | 4126S |  |
| HSL Rabbit pAb | Cell Signaling Technology | 4107S |  |
| PI3 Kinase p85 Rabbit pAb | Cell Signaling Technology | 4292S |  |
| Phospho-AKT (Ser473) (D9E) Rabbit mAb | Cell Signaling Technology | 4060S |  |
| AKT (pan) (C67E7) Rabbit mAb | Cell Signaling Technology | 4691S |  |
| Phospho-GSK-3β (Ser9) (D85E12) Rabbit mAb | Cell Signaling Technology | 5558T |  |
| GSK-3β (D5C5Z) Rabbit mAb | Cell Signaling Technology | 12456T |  |
| Phospho-MEK1/2 (Ser217/221) (41G9) Rabbit mAb | Cell Signaling Technology | 9154S |  |
| MEK1/2 (D1A5) Rabbit mAb | Cell Signaling Technology | 8727S |  |
| Phospho-p44/42 MAPK (ERK1/2) Rabbit mAb | Cell Signaling Technology | 4370S |  |
| p44/42 MAPK (ERK1/2) Rabbit pAb | Cell Signaling Technology | 9102S |  |
| Phospho-PKA C (Thr197) Rabbit pAb | Cell Signaling Technology | 4781S |  |
| PKA C-α Rabbit pAb | Cell Signaling Technology | 4782S |  |
| Phospho-CREB (Ser133) (87G3) Rabbit mAb | Cell Signaling Technology | 9198S |  |
| CREB (48H2) Rabbit mAb | Cell Signaling Technology | 9197 |  |
| Phospho-p38 MAPK (Thr180/Tyr182) Rabbit pAb | Cell Signaling Technology | 9211S |  |
| p38 MAPK Rabbit pAb | Cell Signaling Technology | 9212S |  |
| GAPDH Rabbit pAb | BBI Life Sciences | D110016 |  |
| IRDye 800CW Donkey anti-Mouse IgG (H + L) | LI-COR | 925-32212 |  |
| IRDye 800CW Donkey anti-Rabbit IgG (H + L) | LI-COR | 925-32213 |  |
| Goat anti-Rabbit IgG (H+ L), Alexa Fluor 488 | Invitrogen | A11001 |  |
| Goat anti-Rabbit IgG (H+L) , Alexa Fluor 555 | Invitrogen | A21428 |  |
